# Composite inflammatory biomarkers for predicting vascular crisis after finger replantation: a two-center retrospective study identifying a promising inflammatory biomarker and its multidimensional evaluation

**DOI:** 10.3389/fimmu.2026.1915327

**Published:** 2026-07-29

**Authors:** Yongsheng Tian, Shuai Dong, Yuji Zhang, Zhengjie Li, Zhongzheng Liu, Zefeng Liu, Chao Geng, Liping Guo, Liang Yang, Jiayu Shen, Jihui Ju, Rong Zhou

**Affiliations:** Department of Hand Surgery, Suzhou Ruihua Orthopedic Hospital of Soochow University, Suzhou, China

**Keywords:** composite inflammatory biomarkers, finger replantation, risk prediction, systemic inflammatory response index, vascular crisis

## Abstract

**Background:**

Vascular crisis is a leading cause of replantation failure following finger replantation, and preoperative identification of high-risk patients is essential for improving clinical outcomes. Composite inflammatory biomarkers derived from routine blood tests have demonstrated predictive value across multiple surgical disciplines; however, their role in predicting vascular crisis after finger replantation has not been systematically evaluated.

**Methods:**

This retrospective study enrolled 1, 744 patients who underwent finger replantation, classified into a vascular crisis group (n = 102) and a non-vascular crisis group (n = 1, 642). Eight preoperative composite inflammatory biomarkers were calculated, including SIRI, NPAR, AISI, NLR, SII, MLR, NPR, and PLR. Three stepwise-adjusted logistic regression models were constructed, and the optimal biomarker was identified through ROC curve analysis. Subsequent analyses included restricted cubic spline (RCS) modeling, threshold effect analysis, subgroup analysis, mediation analysis, and multinomial logistic regression. Propensity score matching (PSM) with mixed-effects logistic regression was performed for validation.

**Results:**

The overall incidence of vascular crisis was 5.85%. In the fully adjusted model, the highest tertile groups of SIRI, NPAR, AISI, NLR, SII, and NPR were significantly associated with increased vascular crisis risk (all *P* < 0.050). SIRI demonstrated the highest discriminative ability (AUC = 0.695), with an optimal cutpoint of 1.214. RCS analysis revealed a nonlinear association between SIRI and vascular crisis (*P*-nonlinear < 0.001), with a breakpoint at 3.388, below which each unit increase in SIRI was significantly associated with elevated risk (adjusted OR = 2.00, 95% CI: 1.45 to 2.77, *P* < 0.001). Subgroup analysis demonstrated a stronger predictive effect among smokers (*P* for interaction = 0.018). D-dimer partially mediated the SIRI-vascular crisis association (mediated proportion 10.00%). SIRI exhibited more robust predictive performance for venous than arterial crisis. PSM validation confirmed significantly elevated risk in the high SIRI group (OR = 2.65, 95% CI: 1.81 to 3.88, *P* < 0.001).

**Conclusion:**

Preoperative SIRI was significantly associated with vascular crisis following finger replantation and showed the highest numerical AUC among the tested composite inflammatory biomarkers. SIRI may serve as a supplementary tool for perioperative risk stratification and warrants further validation in two-center prospective studies.

## 1 Introduction

Finger replantation represents a landmark achievement in hand surgery. Since Komatsu and Tamai performed the first successful finger replantation in 1965 (1), continuous advances in microsurgical techniques have elevated the overall survival rate of replanted digits to 70% to 90% (2, 3). Despite these technical improvements, postoperative vascular crisis remains a serious complication following finger replantation and a leading cause of replantation failure (4). Arterial vasospasm and thrombosis are the primary pathological mechanisms underlying replantation failure, and failure to restore timely perfusion can result in irreversible necrosis and ultimate loss of the replanted digit (5).

Previous studies have largely focused on identifying risk factors for poor outcomes after finger replantation. Gao et al. developed a prediction model for replantation failure based on a multicenter prospective cohort of more than 2, 000 replanted digits, identifying six independent predictors of failure including age. The model achieved C-indices of 0.81 and 0.70 in the training and external validation sets, respectively, demonstrating satisfactory predictive performance (6). A systematic review and meta-analysis further reported that clean-cut injuries and a greater number of venous anastomoses were associated with higher survival rates, whereas crush and avulsion injuries were indicative of poorer prognosis (7). However, the predictors examined in these studies are predominantly intraoperative or perioperative clinical variables, with a focus on overall replantation failure rather than vascular crisis specifically. Objective, easily accessible, and preoperatively available laboratory-based predictors remain scarce.

In recent years, composite inflammatory biomarkers derived from routine blood tests, including the neutrophil-to-lymphocyte ratio (NLR), platelet-to-lymphocyte ratio (PLR), systemic immune- inflammation index (SII), and systemic inflammatory response index (SIRI), have attracted considerable attention for their potential to predict postoperative complications across various surgical disciplines. Their advantages include simple calculation, no additional cost, and the ability to comprehensively reflect systemic inflammatory status. Relevant applications include predicting pneumonia after hip fracture surgery (8), infectious complications following posterior spinal instrumentation (9), and short- and long-term prognosis in patients undergoing surgery for acute type A aortic dissection (10). Nevertheless, none of these studies have examined microsurgical reconstruction or finger replantation, and the association between composite inflammatory biomarkers and postoperative vascular crisis after finger replantation has not been systematically investigated. Notably, inflammation has been implicated in both vascular vasospasm (11) and thrombosis formation (12).

Based on the above, this study enrolled 1, 744 patients who underwent finger replantation to systematically compare the predictive performance of eight composite inflammatory biomarkers for postoperative vascular crisis. The optimal biomarker was further evaluated through multidimensional analyses to explore its potential value in preoperative risk assessment among patients undergoing finger replantation.

## 2 Methods

### 2.1 Study design and population

**Figure 1:**
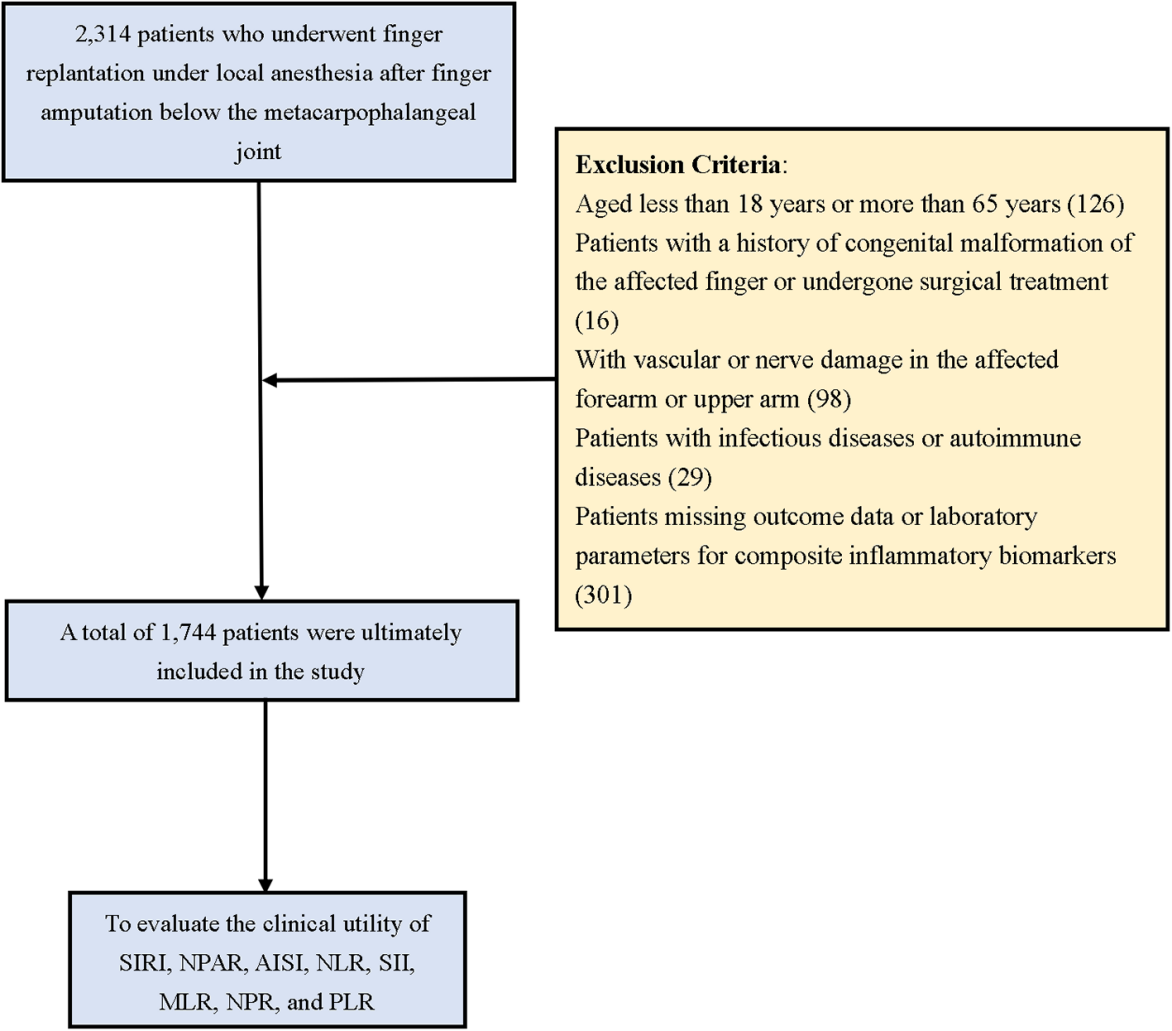
Flowchart of patient selection in the present study.

This two-center retrospective observational study was conducted in accordance with the updated STROCSS 2025 guidelines (13) and retrospectively collected clinical data from young and middle-aged patients who underwent finger replantation surgery following traumatic finger amputation at two hospitals with advanced microsurgical capabilities, namely Suzhou Ruihua Orthopedic Hospital and Suzhou Shengze Hospital, between September 2018 and September 2023. Data collection was independently performed and cross-verified by the co-first authors to ensure data accuracy, with any discrepancies resolved through discussion and consensus. Given the retrospective nature of this study, informed consent from patients was not required. A total of 2, 314 patients who underwent finger replantation under local anesthesia for finger amputation below the metacarpophalangeal joint were initially screened. Patients were excluded according to the following criteria: (1) 126 patients aged less than 18 years or more than 65 years; (2) 16 patients with a history of congenital malformation of the affected finger or who had previously undergone surgical treatment of the affected finger; (3) 98 patients with vascular or nerve damage in the affected forearm or upper arm; (4) 29 patients with infectious diseases or autoimmune diseases; (5) 301 patients with missing laboratory parameters required for calculating composite inflammatory biomarkers or missing outcome data (Missing data primarily resulted from incomplete laboratory examinations or incomplete documentation at the time of admission. Because this was a retrospective study, no imputation was performed, and cases with missing key variables were excluded from the final analysis). Ultimately, 1, 744 patients were included in the final analysis, as illustrated in **Figure 1**.

### 2.2 The definition and calculation of inflammatory indices

All composite inflammatory biomarkers analyzed in this study were calculated based on corresponding peripheral blood parameters. The relevant parameters included neutrophil count (NEU, ×10^9^/L), lymphocyte count (LYM, ×10^9^/L), monocyte count (MONO, ×10^9^/L), platelet count (PLT, ×10^9^/L), neutrophil percentage (NEU%, %) and albumin level (ALB, g/L). Consistent with previously published literature (14–16), the calculation formulas for each biomarker were as follows:


SII=PLT×NEU/LYM

NLR=NEU/LYM

PLR=PLT/LYM

SIRI=NEU×MONO/LYM

Monocyte‐to‐lymphocyte ratio(MLR)=MONO/LYM

Neutrophil‐to‐platelet ratio(NPR)=NEU/PLT

Aggregate index of systemic inflammation(AISI)=NEU×MONO×PLT/LYM

Neutrophil percentage‐to‐albumin ratio (NPAR)=NEU%/ALB

### 2.3 Definition and management of postoperative vascular crisis

Postoperative vascular crisis was defined as the occurrence of arterial insufficiency or venous congestion in the replanted digit during the in-hospital postoperative period. Arterial crisis was characterized by skin pallor, absence of capillary refill, decreased skin temperature, and absence of bleeding upon pulp pressure. Venous crisis was characterized by dark red or cyanotic skin discoloration, increased tissue turgor, and active dark red bleeding upon pulp pressure. When postoperative vascular crisis was suspected, immediate clinical assessment was performed. For arterial crisis, potential external compression was relieved and vasodilatory agents were administered. If circulation did not improve promptly, emergent surgical exploration was undertaken. During re-exploration, the thrombosed arterial segment was respected and reconstructed by direct anastomosis or vein grafting as indicated. For venous crisis, temporary decompressive measures were applied as appropriate, including controlled fingertip bleeding. Cases involving persistent venous dysfunction or proximal digit replantation underwent emergent surgical exploration with venous repair or vein graft reconstruction (4, 17).

### 2.4 Assessment of covariates

Consistent with previous studies (18, 19), to minimize the potential confounding effects of factors influencing the occurrence of vascular crisis, we incorporated the following covariates into the analysis. Basic patient characteristics included age, sex, and smoking history. Common comorbidities included hypertension and diabetes. Perioperative-related factors included preservation method (dry at low temperature, dry at room temperature, or contact with liquid), injured finger (thumb, index, middle, ring, little, or multidigit), injury number, injury level (Tamai I to V or multiple levels), injury mechanism (chainsaw injury, crush injury, avulsion injury, or clean cut injury), bone and joint injury (syntripsis, fracture involving the joint, or transverse fracture), nerve injury (fingertip nerve injury, crush injury of digital nerve, cut injury of digital nerve, or avulsion injury of digital nerve), tendon injury (none, Zone I to V), artery injury (fingertip artery injury, crush injury of digital artery, cut injury of digital artery, avulsion injury of digital artery, or avulsion injury of digital artery with defect ≥1.5 cm), nail bed or dorsal skin injury (nail bed injury, nail bed and dorsal finger skin injury, crush injury of dorsal finger skin, cut injury of dorsal finger skin, avulsion injury of dorsal finger skin, or avulsion injury with defect of dorsal finger skin), ischemic time, duration of surgery, surgeon seniority [senior surgeons were defined as those who had performed 50 or more finger replantation procedures during their career, while junior surgeons had performed 49 or fewer (20)], utilization of vein graft (yes or no), number of arteries repaired, number of veins repaired, and flap coverage (yes or no). Peripheral venous blood samples were routinely collected from all patients immediately after admission to our hospital, before surgery and prior to the administration of perioperative medications. All laboratory parameters included in this study were obtained from the first blood test performed after admission at our institution. As finger replantation is an emergency procedure, these laboratory measurements were collected after injury but before surgery, thereby reflecting the patients’ preoperative inflammatory status at the time of clinical evaluation. Laboratory parameters included hemoglobin (Hb) level, ALB level, D-dimer level, PLT count, NEU count, LYM count, MONO count, and NEU%.

### 2.5 Statistical analysis

All statistical analyses were performed using R software (version 4.4.1). Continuous variables were expressed as mean ± standard deviation (SD) or median and interquartile range (IQR) according to their distribution and compared between groups using the independent samples t-test or Mann-Whitney U test based on normality assessment. Categorical variables were expressed as frequencies and percentages and compared using the chi-square test or Fisher’s exact test. Baseline characteristics were summarized according to the occurrence of postoperative vascular crisis, and boxplots were used to visualize the distribution of composite inflammatory biomarkers between the vascular crisis and non-vascular crisis groups.

Three stepwise-adjusted binary logistic regression models were constructed to evaluate the associations between composite inflammatory biomarkers and the risk of postoperative vascular crisis. Each biomarker was incorporated into the models both as a continuous variable and as a categorical variable grouped by tertiles, to simultaneously capture linear trends and stratified effects. Model 1 was unadjusted. Model 2 was adjusted for age, sex, and smoking history. Model 3 was further adjusted for perioperative-related factors and non-calculated laboratory parameters based on Model 2. Regression results were expressed as odds ratios (OR) with 95% confidence intervals (CI) and were visually presented using forest plots to illustrate effect estimates and their uncertainty across different adjustment models. Receiver operating characteristic (ROC) curve analysis was subsequently performed to compare the overall discriminative ability of each composite inflammatory biomarker for vascular crisis, and the biomarker with the highest area under the curve (AUC) was selected for all subsequent analyses. A restricted cubic spline (RCS) model was applied to examine the potential nonlinear dose-response relationship between the optimal biomarker and vascular crisis. Subgroup analyses were conducted to assess the consistency of the association across populations with different clinical characteristics, and potential effect modification was formally evaluated using likelihood ratio tests for interaction terms. Following identification of the optimal predictive biomarker, its mediating role in the pathway between composite inflammatory markers and vascular crisis was further explored through mediation analysis to quantify the proportion of direct and indirect effects and elucidate the underlying biological mechanisms. In addition, a three-category outcome analysis (no crisis, venous crisis, and arterial crisis) was performed with no crisis as the reference category, and multinomial logistic regression was used to independently estimate the predictive effects of composite inflammatory biomarkers on venous crisis and arterial crisis. Three stepwise-adjusted models were similarly constructed, and the predictive advantage of the biomarker for different types of vascular crisis was evaluated by comparing the corresponding OR values and 95% CIs, thereby enabling differentiated prediction across crisis subtypes.

Finally, participants were divided into low- and high-level groups according to the optimal cutoff value determined by ROC analysis. Propensity score matching (PSM) was subsequently performed using 1:1 nearest-neighbor matching, without replacement, with a caliper width of 0.20 to balance baseline characteristics and reduce potential confounding bias (21). The propensity score model included all covariates described above, except for the laboratory variables used to calculate the composite inflammatory biomarker. Covariate balance after matching was assessed using standardized mean differences (SMDs), with an SMD < 0.10 considered indicative of adequate balance between the two groups. In the matched cohort, a mixed-effects logistic regression model was fitted to evaluate the association between biomarker categories and vascular crisis while accounting for the within-pair correlation introduced by the PSM process. All statistical tests were two-sided, and a *P* value < 0.050 was considered statistically significant.

## 3 Results

### 3.1 Baseline characteristics and distribution of composite inflammatory biomarkers

**Figure 2:**
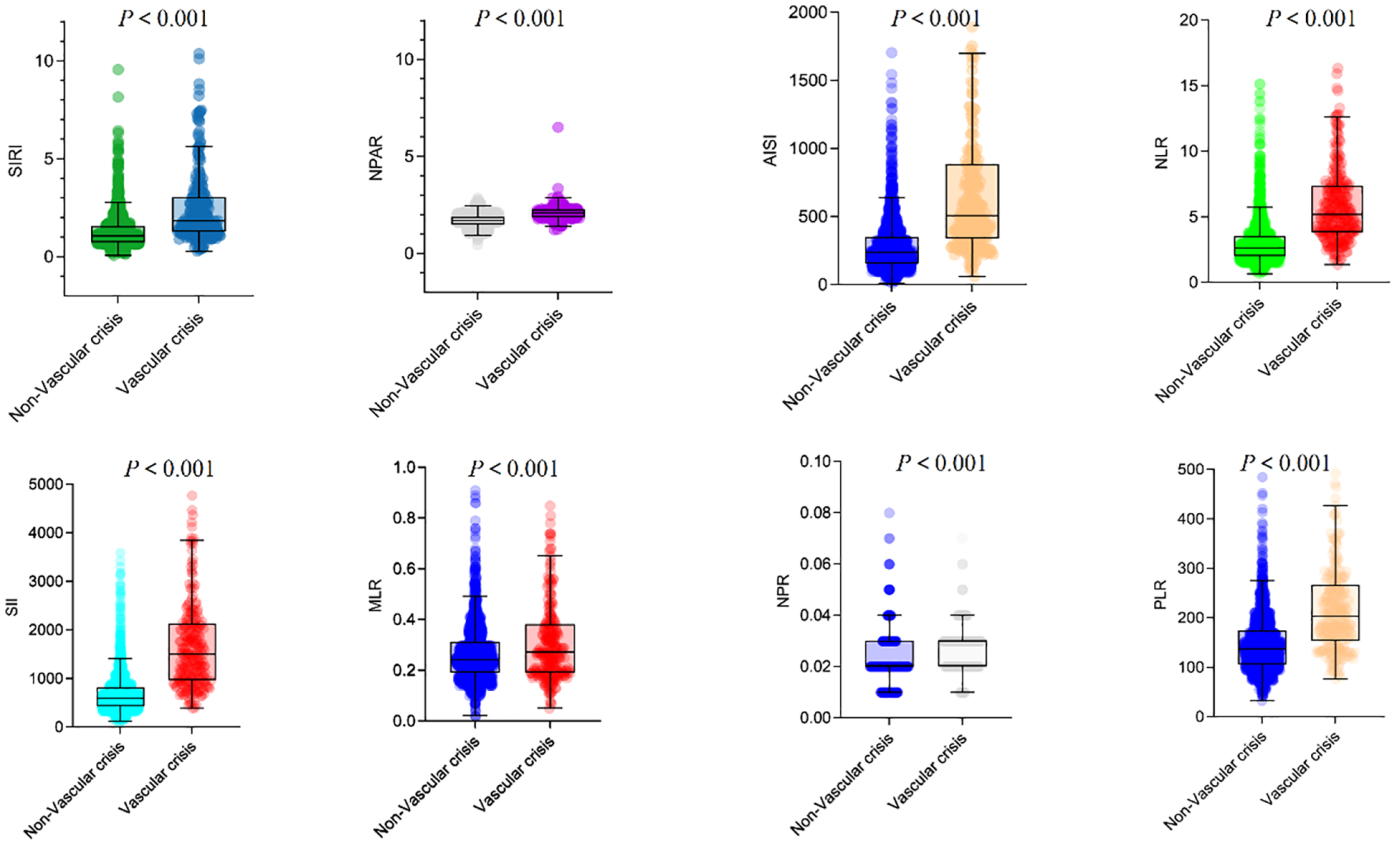
Distribution of composite inflammatory biomarkers between non-vascular crisis and vascular crisis groups presented as boxplots.

A total of 1, 744 patients who underwent finger replantation were enrolled in this study, comprising 1, 642 patients in the non-vascular crisis group (94.15%) and 102 patients in the vascular crisis group (5.85%). Baseline characteristics of both groups are summarized in **Supplementary Table 1**. No statistically significant differences were observed between the two groups in terms of age, ischemic time, duration of surgery, complications, injured finger, injury number, bone and joint injury, nerve injury, tendon injury, number of veins repaired, arteriovenous ratio, or skin injury (all *P* > 0.050). In contrast, statistically significant differences were identified between the two groups with respect to smoking history, hypertension, preservation method, surgeon seniority, injury mechanism, utilization of vein graft, artery injury type, number of arteries repaired, and flap coverage (all *P* < 0.050). All eight composite inflammatory biomarkers (SIRI, NPAR, AISI, NLR, SII, MLR, NPR, and PLR) were significantly elevated in the vascular crisis group compared with the non-vascular crisis group, and the differences between groups were statistically significant for all biomarkers (all *P* < 0.001). The distribution of each biomarker across the two groups is illustrated in **Figure 2**.

### 3.2 Associations between composite inflammatory biomarkers and vascular crisis risk: stepwise-adjusted logistic regression analysis

**Figure 3:**
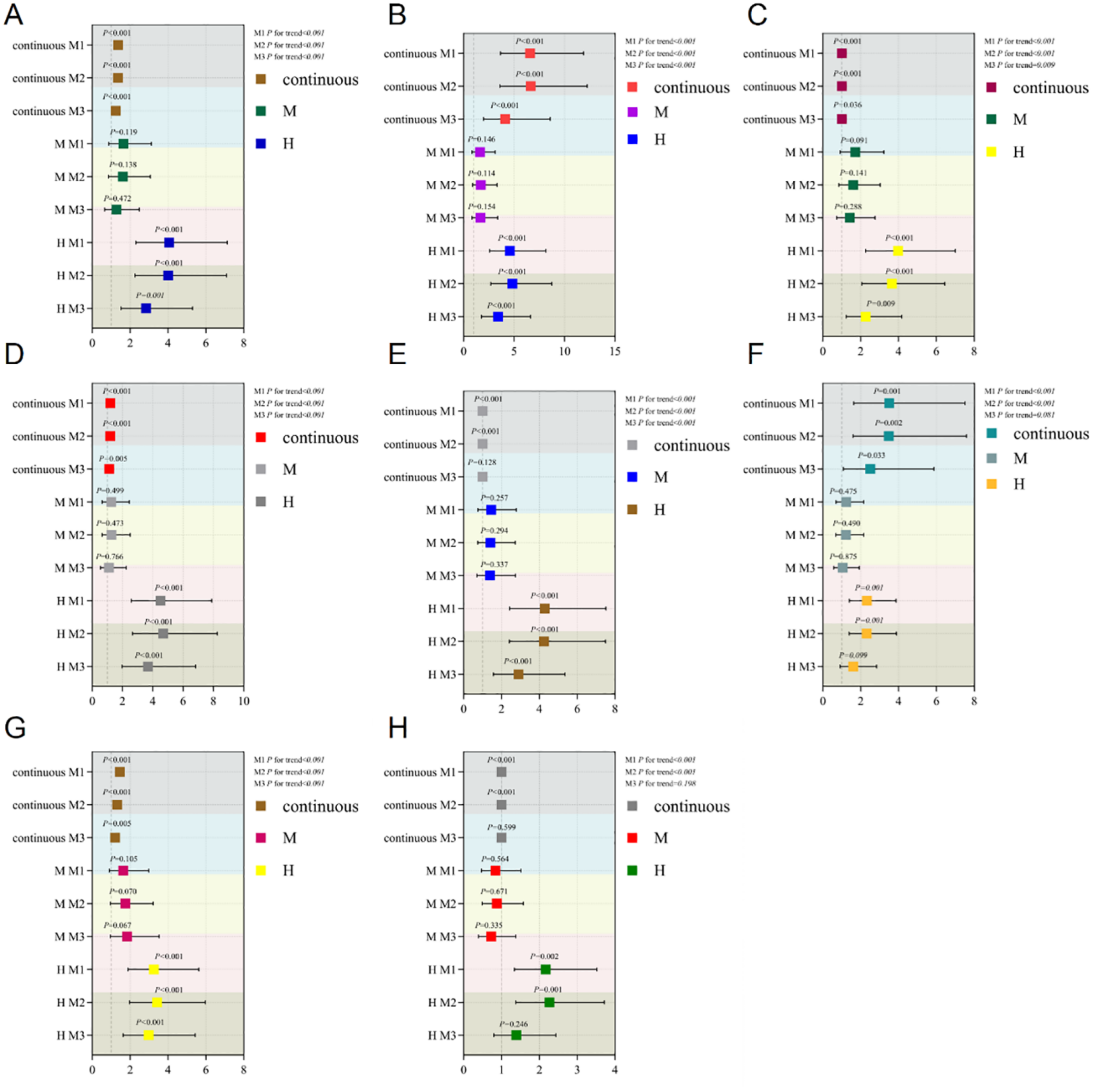
Forest plots of the associations between composite inflammatory biomarkers and vascular crisis risk across three stepwise-adjusted logistic regression models. **(A)** SIRI; **(B)** NPAR; **(C)** AISI; **(D)** NLR; **(E)** SII; **(F)** MLR; **(G)** NPR; **(H)** PLR. M1, unadjusted model; M2, adjusted for sex, age, and smoking; M3, fully adjusted model.

Three stepwise-adjusted logistic regression models were constructed with each biomarker incorporated as both a continuous variable and a tertile-based categorical variable. When analyzed as continuous variables, SIRI, NPAR, AISI, NLR, MLR, and NPR were significantly associated with vascular crisis risk across all three models (all *P* < 0.001), whereas SII and PLR showed statistically significant associations in Model 1 and Model 2 but demonstrated attenuated associations in the fully adjusted Model 3 (*P* > 0.050). In the tertile analysis, using the lowest tertile group (L group) as the reference, patients in the highest tertile group (H group) in the fully adjusted Model 3 showed significantly elevated risks of vascular crisis for SIRI (OR = 2.84, 95% CI: 1.52 to 5.30), NPAR (OR = 3.41, 95% CI: 1.76 to 6.62), AISI (OR = 2.27, 95% CI: 1.23 to 4.17), NLR (OR = 3.68, 95% CI: 1.98 to 6.84), and NPR (OR = 2.98, 95% CI: 1.63 to 5.44), all of which reached statistical significance (all *P* < 0.050), with statistically significant trends across tertiles (all *P* for trend < 0.050). The trend test for MLR was statistically significant in Models 1 and 2 but was not statistically significant in Model 3(*P =* 0.081). Detailed results are presented in **Figure 3**.

### 3.3 Discriminative performance of composite inflammatory biomarkers for vascular crisis: ROC curve analysis and optimal biomarker selection

**Figure 4:**
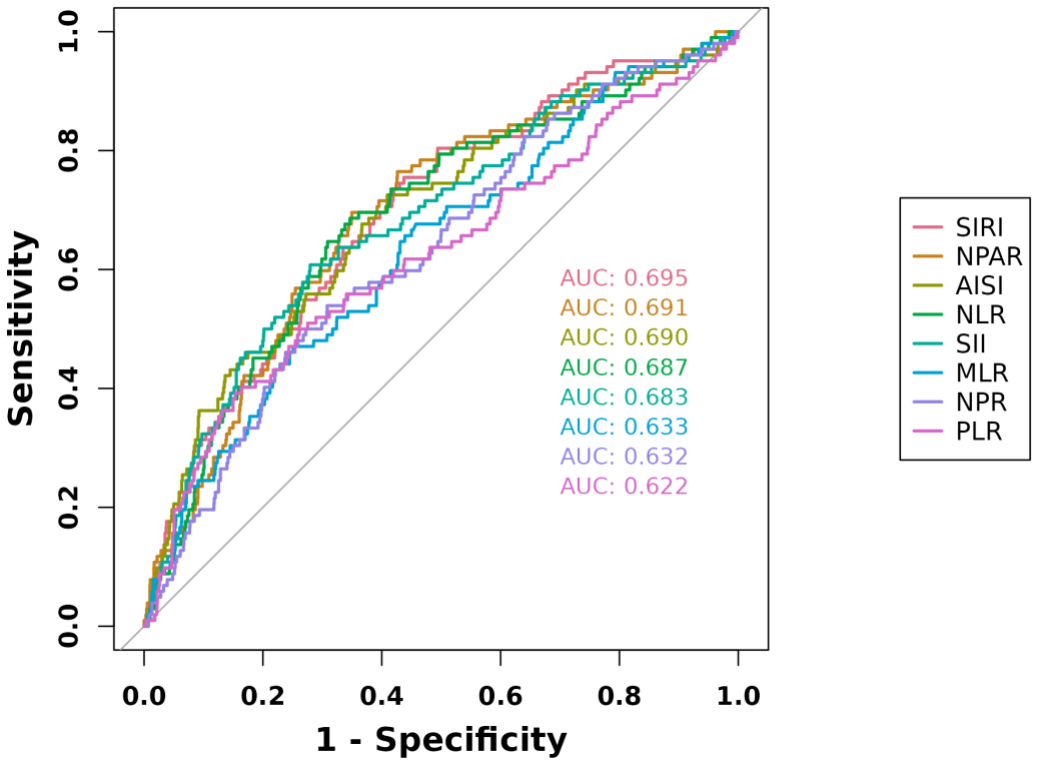
ROC curves of eight composite inflammatory biomarkers for predicting vascular crisis following finger replantation.

ROC curve analysis was performed to evaluate the discriminative ability of all eight composite inflammatory biomarkers for predicting vascular crisis. SIRI achieved the highest AUC (AUC = 0.695), followed by NPAR (AUC = 0.691), AISI (AUC = 0.690), NLR (AUC = 0.687), SII (AUC = 0.683), MLR (AUC = 0.633), NPR (AUC = 0.632), and PLR (AUC = 0.622), as illustrated in **Figure 4**. Based on optimal cutpoint analysis, the cutpoint for SIRI was 1.214 (≥1.214), corresponding to a sensitivity of 75.50%, specificity of 56.30%, positive predictive value (PPV) of 9.70%, negative predictive value (NPV) of 97.40%, accuracy of 57.40%, and a Youden index of 0.318, as detailed in **Supplementary Table 2**. Accordingly, SIRI was selected as the primary biomarker for all subsequent analyses.

### 3.4 Dose-response relationship and threshold effect between SIRI and vascular crisis: RCS and piecewise logistic regression analysis

**Figure 5:**
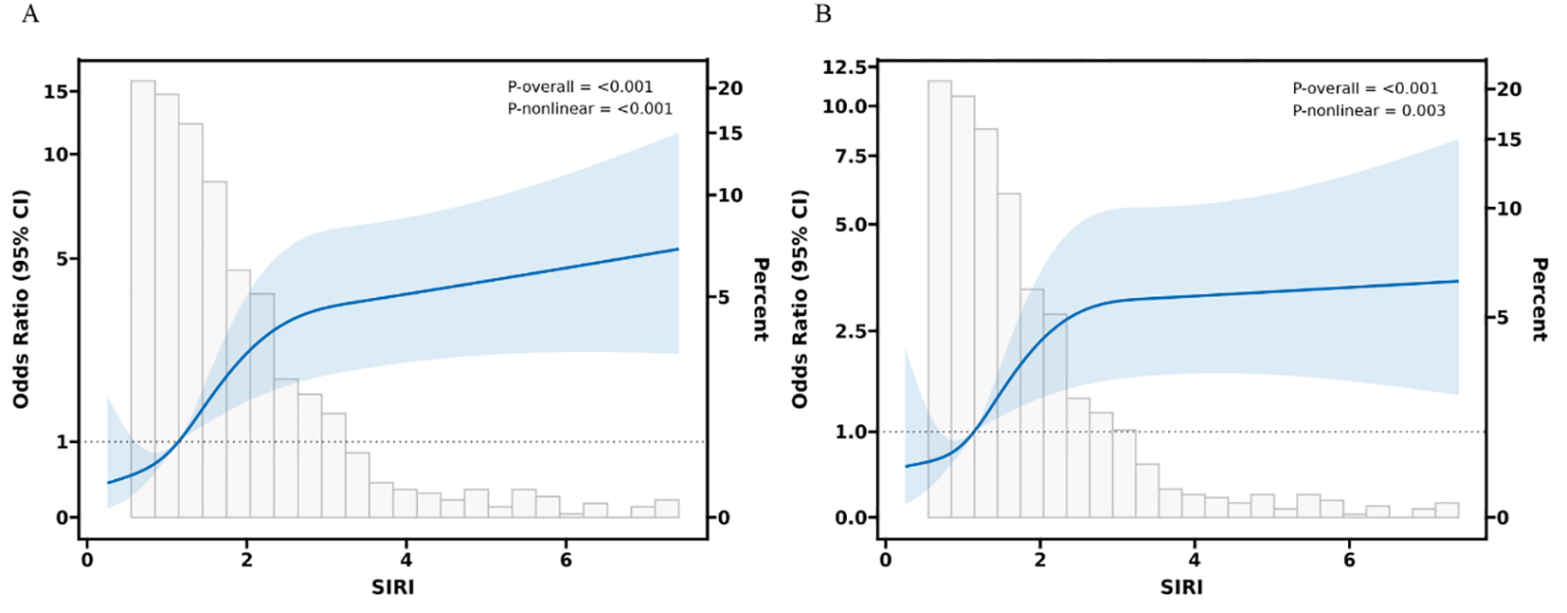
RCS analysis of the association between SIRI and vascular crisis risk. **(A)** Unadjusted model; **(B)** Model adjusted for all covariates.

RCS analysis revealed a significant nonlinear association between SIRI and the risk of vascular crisis (*P*-overall < 0.001, *P*-nonlinear < 0.001). This nonlinear association remained robust in both the unadjusted model (**Figure 5A**) and the model adjusted for all covariates (**Figure 5B**, *P*-overall < 0.001, *P*-nonlinear = 0.003), indicating that the predictive effect of SIRI on vascular crisis does not follow a simple linear pattern.

Threshold effect analysis demonstrated that the piecewise logistic regression model provided a significantly better fit than the standard linear logistic regression model (log likelihood ratio test *P* = 0.001), with a breakpoint identified at SIRI = 3.388. When SIRI was below 3.388, each unit increase in SIRI was associated with a significantly elevated risk of vascular crisis (adjusted OR = 2.00, 95% CI: 1.45 to 2.77, *P* < 0.001). When SIRI was 3.388 or above, the association between SIRI and vascular crisis risk was no longer statistically significant (adjusted OR = 0.91, 95% CI: 0.67 to 1.24, *P* = 0.564), suggesting a saturation effect in the predictive value of SIRI for vascular crisis. Detailed results are presented in **Supplementary Table 3**.

### 3.5 Subgroup analysis of the association between SIRI and vascular crisis risk

**Figure 6:**
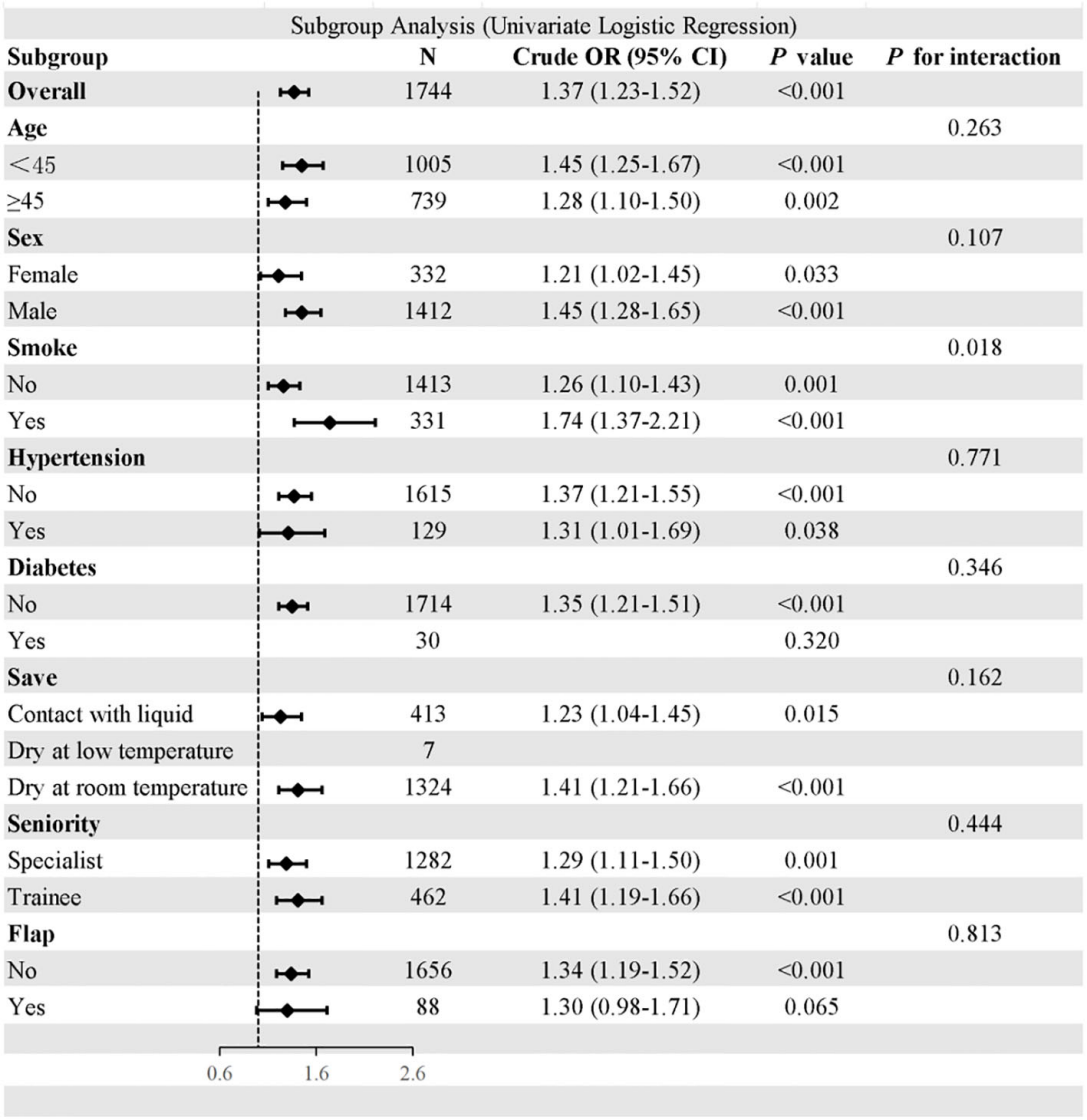
Forest plot of subgroup analyses examining the association between SIRI and vascular crisis risk across clinically relevant strata.

Subgroup analyses demonstrated that the positive association between SIRI and vascular crisis risk was generally consistent across all clinical subgroups (**Figure 6**). SIRI was significantly associated with vascular crisis risk across subgroups defined by age (younger than 45 years: OR = 1.45, 95% CI: 1.25 to 1.67; 45 years or older: OR = 1.28, 95% CI: 1.10 to 1.50), sex, hypertension, diabetes, preservation method, surgeon seniority, and flap coverage, with no statistically significant interaction effects observed (all *P* for interaction > 0.050), indicating that these factors did not significantly modify the association between SIRI and vascular crisis.

Notably, the interaction test in the smoking subgroup reached statistical significance (*P* for interaction = 0.018), with a larger effect size of SIRI observed among smokers (OR = 1.74, 95% CI: 1.37 to 2.21), suggesting that smoking may exert a certain degree of effect modification on the predictive relationship between SIRI and vascular crisis.

### 3.6 Mediation analysis: D-dimer as a mediator in the association between SIRI and vascular crisis

**Figure 7:**
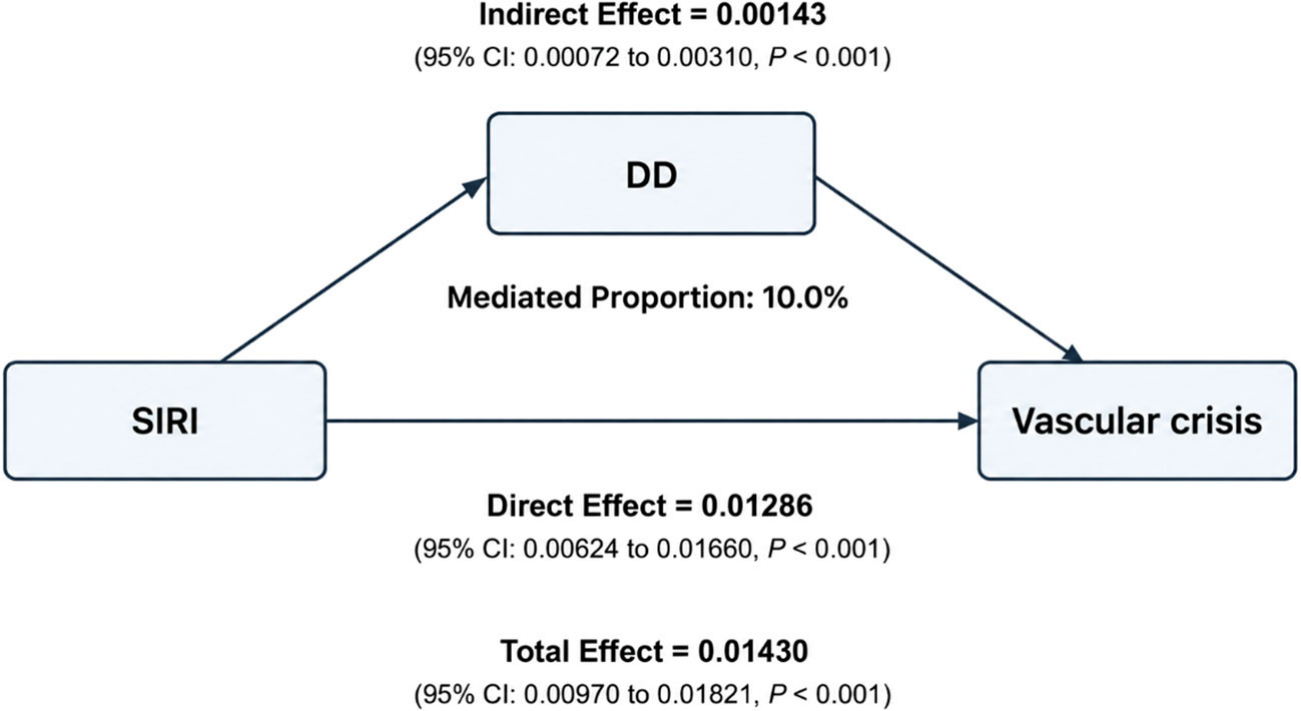
Mediation analysis diagram illustrating the role of D-dimer as a mediator in the association between SIRI and vascular crisis.

Mediation analysis revealed that the total effect of SIRI on vascular crisis was statistically significant (Total Effect = 0.01430, 95% CI: 0.00970 to 0.01821, *P* < 0.001). After controlling relevant covariates, SIRI exerted a significant indirect effect mediated through D-dimer (Indirect Effect = 0.00143, 95% CI: 0.00072 to 0.00310, *P* < 0.001), with D-dimer accounting for a mediated proportion of 10.0%. The direct effect of SIRI on vascular crisis was also statistically significant (Direct Effect = 0.01286, 95% CI: 0.00824 to 0.01660, *P* < 0.001), indicating that the predictive effect of SIRI on vascular crisis is partially mediated through D-dimer, while the direct effect remains predominant, as illustrated in **Figure 7**.

### 3.7 Differential predictive value of SIRI for arterial and venous crisis: multinomial logistic regression analysis

Using the non-vascular crisis group as the reference category, multinomial logistic regression was performed to independently evaluate the predictive effects of SIRI on arterial crisis and venous crisis. SIRI was significantly associated with the risk of both arterial crisis and venous crisis across all three stepwise-adjusted models. For arterial crisis, each unit increase in SIRI was associated with a 35% increase in the risk of arterial crisis in the fully adjusted Model 3 (OR = 1.35, 95% CI: 1.03 to 1.77, P = 0.030). For venous crisis, the predictive effect of SIRI was more pronounced and robust, with an OR of 1.26 in Model 3 (95% CI: 1.11 to 1.43, P < 0.001), and the effect estimates remained highly consistent across all three models. Comparison of effect sizes between the two crisis subtypes indicated that the predictive association of SIRI with venous crisis demonstrated superior statistical robustness compared with arterial crisis, as evidenced by narrower confidence intervals and smaller P values, suggesting that SIRI holds more stable predictive value for venous crisis. Detailed results are presented in **Table 1**.

**Table 1:** Multinomial logistic regression analysis of the association between SIRI and vascular crisis subtypes.

Characteristic	Model 1			Model 2			Model 3		
	OR	95% CI	P-value	OR	95% CI	P-value	OR	95% CI	P-value
Non-Venous crisis	–	–	–	–	–	–	–	–	–
SIRI Arterial crisis	1.37	1.07, 1.74	0.011	1.37	1.07, 1.77	0.014	1.35	1.03, 1.77	0.030
SIRI Venous crisis	1.37	1.22, 1.53	<0.001	1.36	1.21, 1.53	<0.001	1.26	1.11, 1.43	<0.001

CI, Confidence Interval; OR, Odds Ratio.

Model 1: no covariates were adjusted.

Model 2: adjusted for sex, age, and smoke.

Model 3: adjusted for sex, age, Ischemic time, preservation method, complication, duration of surgery, seniority, smoke, hypertension, diabetes, vein graft, degree of detachment, bone and joint, flap and non-calculated laboratory parameters.

### 3.8 Validation of the association between SIRI and vascular crisis following PSM: mixed-effects logistic regression analysis

Based on the optimal cutpoint identified by ROC analysis (SIRI = 1.214), the 1, 744 patients were divided into a high SIRI group (n = 950) and a low SIRI group (n = 794). A 1:1 propensity score matching with a caliper of 0.20 was performed, yielding 723 patients in each group after matching. Prior to matching, the two groups showed significant differences in smoking history, ischemic time, duration of surgery, platelet count, D-dimer level, preservation method, surgeon seniority, and flap coverage (all *P* < 0.050). After matching, the SMDs of all covariates were substantially reduced (all SMDs < 0.10), indicating adequate covariate balance between the two groups. The distribution of all covariates was also well balanced (all *P* > 0.050). Covariate balance before and after matching is summarized in **Table 2** and illustrated in **Supplementary Figure 1**.

**Table 2:** Baseline covariate balance before and after PSM.

Variables	Before matching				After matching			
	high	low	SMD	P-value	high	low	SMD	P-value
**n**	950	794			723	723		
**Gender**				0.014				0.780
Male	749 (78.80)	663 (83.50)	0.126		601 (83.10)	597 (82.60)	-0.015	
Female	201 (21.20)	131 (16.50)	-0.126		122 (16.90)	126 (17.40)	0.015	
**Age (y)**	40.82 (11.65)	40.94 (11.69)	0.010	0.828	40.51 (11.52)	40.98 (11.67)	0.040	0.449
**Smoke**				0.001				0.791
No	796 (83.80)	617 (77.70)	-0.146		583 (80.60)	579 (80.10)	-0.013	
Yes	154 (16.20)	177 (22.30)	0.146		140 (19.40)	144 (19.90)	0.013	
**Hypertension**				0.182				0.606
No	887 (93.40)	728 (91.70)	-0.061		675 (93.40)	670 (92.70)	-0.025	
Yes	63 (6.60)	66 (8.30)	0.061		48 (6.60)	53 (7.30)	0.025	
**Diabetes**				0.540				>0.999
No	932 (98.10)	782 (98.50)	0.031		711 (98.30)	711 (98.30)	0.000	
Yes	18 (1.90)	12 (1.50)	-0.031		12 (1.70)	12 (1.70)	0.000	
**Ischemic time (h)**	5.23 (1.90)	5.58 (2.09)	0.167	<0.001	5.39 (2.00)	5.46 (1.97)	0.034	0.342
**Duration of surgery (h)**	3.49 (2.01)	3.82 (2.15)	0.150	<0.001	3.66 (2.07)	3.68 (2.02)	0.009	0.605
**Hb (g/L)**	137.15 (17.92)	136.44 (17.47)	-0.040	0.256	138.05 (17.40)	137.46 (16.72)	-0.033	0.279
PLT (*109/L)	227.37 (51.95)	244.31 (64.37)	0.263	<0.001	235.03 (51.37)	236.94 (55.72)	0.030	0.686
**D-dimer (mg/L)**	0.30 (0.48)	0.43 (0.60)	0.207	<0.001	0.31 (0.33)	0.33 (0.30)	0.038	0.068
**Preservation method**				0.005				0.792
Dry at low temperature	3 (0.30)	4 (0.50)	0.027		2 (0.30)	3 (0.40)	0.020	
Dry at room temperature	749 (78.80)	575 (72.40)	-0.144		549 (75.90)	541 (74.80)	-0.025	
Contact with liquid	198 (20.80)	215 (27.10)	0.140		172 (23.80)	179 (24.80)	0.022	
**Complication**				0.050				0.475
No	814 (85.70)	653 (82.20)	-0.090		611 (84.50)	601 (83.10)	-0.036	
Yes	136 (14.30)	141 (17.80)	0.090		112 (15.50)	122 (16.90)	0.036	
**Seniority**				<0.001				0.769
Trainee	219 (23.10)	243 (30.60)	0.164		197 (27.20)	202 (27.90)	0.015	
Specialist	731 (76.90)	551 (69.40)	-0.164		526 (72.80)	521 (72.10)	-0.015	
**Vein graft**				0.224				0.860
No	931 (98.00)	771 (97.10)	-0.053		707 (97.80)	706 (97.60)	-0.008	
Yes	19 (2.00)	23 (2.90)	0.053		16 (2.20)	17 (2.40)	0.008	
**Degree of detachment**				0.585				0.769
No	37 (3.90)	27 (3.40)	-0.027		23 (3.20)	25 (3.50)	0.015	
Yes	913 (96.10)	767 (96.60)	0.027		700 (96.80)	698 (96.50)	-0.015	
**Bone and joint**				0.120				0.966
Syntripsis	273 (28.70)	194 (24.40)	-0.100		190 (26.30)	186 (25.70)	-0.013	
Fracture involving the joint	212 (22.30)	194 (24.40)	0.049		172 (23.80)	175 (24.20)	0.010	
Transverse fracture	465 (48.90)	406 (51.10)	0.044		361 (49.90)	362 (50.10)	0.003	
**Flap**				0.009				0.801
No	914 (96.20)	742 (93.50)	-0.112		691 (95.60)	689 (95.30)	-0.011	
Yes	36 (3.80)	52 (6.50)	0.112		32 (4.40)	34 (4.70)	0.011	

In the matched cohort, mixed-effects logistic regression analysis demonstrated that the high SIRI group had a significantly elevated risk of vascular crisis compared with the low SIRI group (OR = 2.65, 95% CI: 1.81 to 3.88, *P* < 0.001), further confirming the robustness of the predictive value of SIRI for vascular crisis. Detailed results are presented in **Supplementary Table 4**.

## 4 Discussion

This study systematically evaluated the associations between eight composite inflammatory biomarkers and the risk of postoperative vascular crisis in a two-center dataset of 1, 744 patients who underwent finger replantation. After stepwise adjustment for confounding factors, SIRI, NPAR, AISI, NLR, and NPR were all significantly associated with vascular crisis risk, whereas the associations of SII, MLR and PLR attenuated in the fully adjusted model. ROC curve analysis indicated that SIRI had the highest discriminative ability (AUC = 0.695), and it demonstrated robust and consistent predictive effects across subsequent RCS analysis, threshold effect analysis, subgroup analysis, mediation analysis, and PSM validation. Furthermore, multinomial logistic regression showed that SIRI was significantly associated with both venous and arterial crisis, with a stronger association observed for venous crisis; therefore, SIRI may serve as a supplementary tool for perioperative vascular crisis risk stratification rather than a standalone predictor.

Composite inflammatory biomarkers have been widely validated as predictive tools for postoperative complications across multiple surgical disciplines. Zhu et al. demonstrated in a large-scale cohort of 136, 347 surgical patients that elevated preoperative NLR was independently associated with in-hospital mortality and ICU admission across multiple surgical subgroups (22). In vascular surgery, Drăgan et al. reported that postoperative NLR and SIRI were significantly associated with in-hospital mortality (23). Yücel et al. demonstrated that SIRI and MLR are promising early biomarkers in postoperative pericardiotomy syndrome (24). In microsurgical reconstruction, Zheng et al. identified key clinical predictors of vascular crisis in free flap patients (25). In the context of finger replantation, multiple studies have confirmed the role of D-dimer and inflammatory markers in postoperative outcomes (26–28). Nevertheless, systematic evaluation of composite inflammatory biomarkers targeting vascular crisis specifically in finger replantation remains scarce. In recent years, growing evidence has highlighted the importance of integrating inflammatory biomarkers into broader frameworks of precision medicine and causal inference. Advances in genetic and multi-omics approaches have enabled more refined risk stratification models and individualized prediction strategies for cardiovascular diseases (29). In parallel, Mendelian randomization and combined observational-genetic study designs have strengthened the evidence for potential causal relationships between inflammatory biomarkers and disease susceptibility (30). These developments further support the translational value of biomarker-based approaches in vascular and inflammatory conditions. Based on this background, this study performs a systematic head-to-head comparison of eight composite inflammatory biomarkers and confirms the predictive superiority of SIRI through multidimensional analyses, providing supplementary evidence for perioperative risk stratification in this population.

SIRI showed relatively higher numerical predictive performance among the evaluated composite inflammatory biomarkers, which may be partly related to its unique biological composition. SIRI integrates neutrophils, monocytes, and lymphocytes, thereby reflecting the balance between innate inflammatory activation and adaptive immune regulation. Compared with platelet-containing indices such as SII and AISI, SIRI incorporates monocytes, which play a central role in endothelial inflammation, ischemia–reperfusion injury, and thrombo-inflammatory responses following tissue injury (31). This biological characteristic may render SIRI more closely associated with the pathophysiological processes underlying vascular crisis after finger replantation. Consistent with our findings, Mangalesh et al. reported that SIRI outperformed both NLR and MLR in predicting major adverse cardiovascular events in patients with acute coronary syndrome (32). Although NPAR and AISI also demonstrated predictive value, their effect sizes after full adjustment were slightly lower than that of SIRI, further supporting the potential relevance of monocyte-related inflammation in the development of vascular crisis. In the subgroup analysis, the association between SIRI and vascular crisis was generally consistent across most clinical subgroups. A statistically significant interaction was observed in the smoking subgroup, with a stronger association between SIRI and vascular crisis among smokers. This finding may be biologically plausible, as smoking induces endothelial dysfunction and systemic inflammation through multiple pathways, including upregulation of ICAM-1 and VCAM-1, increased leukocyte adhesion and transmigration, and reduced nitric oxide bioavailability, thereby potentially amplifying inflammatory and pro-thrombotic responses (33–35). However, it should be noted that the subgroup analyses were based on unadjusted models, and some subgroups contained relatively small sample sizes, which may lead to unstable estimates. Therefore, these findings should be interpreted with caution and considered exploratory in nature. Further studies using fully adjusted interaction models and larger sample sizes are required to validate these observations.

Mediation analysis indicated that D-dimer partially mediates the association between SIRI and vascular crisis, accounting for 10.00% of the total effect. However, this finding should be interpreted as exploratory, given the retrospective design and the fact that both SIRI and D-dimer were derived from preoperative or admission laboratory measurements, which limits the ability to establish a clear temporal sequence and causal inference. From a biological perspective, elevated SIRI reflects excessive activation of neutrophils and monocytes, which initiate pro-coagulant cascades through neutrophil extracellular trap (NET) formation, activating the intrinsic coagulation pathway, upregulating tissue factor (TF) expression, and ultimately elevating D-dimer levels (36). As a fibrin degradation product, elevated D-dimer indicates increased microthrombosis risk at the anastomotic site (37), thereby precipitating vascular crisis. The mediated proportion of only 10.0%, however, suggests that direct inflammatory injury to the vascular anastomosis, including endothelial damage, vasospasm, and microcirculatory dysfunction, likely constitutes the predominant mechanism and warrants further investigation. Multinomial logistic regression demonstrated that SIRI was significantly associated with both venous and arterial crisis, with a more robust association observed for venous crisis (Model 3: OR = 1.26, *P* < 0.001) than for arterial crisis (Model 3: OR = 1.35, *P* = 0.030), although the arterial crisis subgroup showed wider confidence intervals. This apparent difference may partly reflect biological differences between the two types of vascular compromise. Consistent with previous microsurgical studies, venous thrombosis is the leading cause of flap or digit failure and occurs more than twice as frequently as arterial thrombosis (38). The slow blood flow, low shear stress, and blood stasis within the venous system may act synergistically with inflammation-induced hypercoagulability to promote venous thrombus formation, whereas arterial crisis is more strongly influenced by vasospasm, anastomotic tension, and local hemodynamic factors (4). In addition, inflammation and thrombosis interact through immunothrombosis and thromboinflammatory pathways (39), providing a biological rationale for the observed association. However, the relatively small number of arterial crisis cases in the present study may also have limited the statistical power to detect comparable associations. Therefore, the observed difference between venous and arterial crisis may reflect both biological specificity and limited statistical power, and should be interpreted cautiously until confirmed in larger prospective studies.

The findings of this study have practical implications for perioperative management of finger replantation. SIRI is derived from routine blood tests, requires no additional cost, and is readily available in the perioperative period, making it feasible for implementation in both primary and specialized surgical centers. Clinicians may calculate SIRI upon patient admission and use it to inform surgical planning, postoperative monitoring intensity, and nursing resource allocation. Given the superior predictive value of SIRI for venous crisis, postoperative surveillance in high SIRI patients may be appropriately directed toward signs of venous outflow obstruction, including digital swelling, skin color changes, and capillary refill time. Additionally, given the larger effect size of SIRI observed among smokers, more proactive vascular crisis surveillance may be warranted in high SIRI patients with a smoking history. It should be noted that the AUC of 0.695 reflects moderate discriminative ability and given the emergent nature of finger replantation surgery with its limited preoperative assessment window, SIRI should serve as a supplementary reference rather than a standalone decision-making tool. Early recognition of vascular crises remain primarily dependent on continuous postoperative clinical observation, and SIRI is best utilized as one component of preoperative risk stratification to assist clinical teams in prioritizing high-risk patients under resource-limited conditions. Prospective studies are needed to further validate the clinical impact of SIRI-guided intervention strategies on patient outcomes.

### Strengths and limitations

This study has several strengths. First, the relatively large sample size of 1, 744 patients who underwent finger replantation provides adequate statistical power. Second, this study systematically compared the predictive performance of eight composite inflammatory biomarkers for vascular crisis in a comprehensive head-to-head design. Third, a multidimensional analytical framework was employed, encompassing stepwise-adjusted logistic regression, RCS nonlinear analysis, threshold effect analysis, mediation analysis, multinomial logistic regression, and PSM validation, thereby providing multi-perspective verification of the robustness of the findings. Fourth, SIRI is derived from routine blood tests and is highly accessible in clinical practice, conferring considerable potential for translational application.

However, this study has several limitations. First, the retrospective design, despite the application of PSM and other methods to control for confounding, precludes the complete exclusion of unmeasured confounding factors inherent to this study design. Second, the number of arterial crisis cases was relatively small (n = 12) compared with venous crisis cases (n = 90), resulting in limited statistical power for this subgroup, which may partly explain the wider confidence intervals and relatively lower robustness of the predictive effect of SIRI for arterial crisis. Validation in larger samples is needed in future studies. In addition, a total of 301 patients were excluded due to missing laboratory or outcome data, which may introduce potential selection bias.

## 5 Conclusions

This study found that preoperative SIRI was significantly associated with vascular crisis following finger replantation and showed the highest numerical predictive performance among the evaluated composite inflammatory biomarkers. D-dimer may partially mediate this association, and the predictive association of SIRI appeared to be stronger for venous crisis than for arterial crisis. As a readily accessible indicator derived from routine blood tests, SIRI may serve as a supplementary reference tool for perioperative risk stratification in finger replantation, although its clinical utility warrants further validation through multicenter prospective studies.

## Data Availability

The raw data supporting the conclusions of this article will be made available by the authors, without undue reservation.
