# Supplementary material for: Composite inflammatory biomarkers for predicting vascular crisis after finger replantation: a two-center retrospective study identifying a promising inflammatory biomarker and its multidimensional evaluation
Source: Front Immunol. 2026 Jul 29;17:1915327. doi: 10.3389/fimmu.2026.1915327 (PMC13481878; doi:10.3389/fimmu.2026.1915327)
Supplement: Supplementary file 1 [file DataSheet1.docx]

**Supplementary Figure 1. Covariate balance before and after PSM**

**Supplementary Table 1. Baseline characteristics of patients stratified by vascular crisis status following finger replantation**

**Supplementary Table 2. Optimal cutpoint analysis of SIRI for predicting vascular crisis**

**Supplementary Table 3. Threshold effect analysis of SIRI on vascular crisis using standard and piecewise logistic regression models, with identification of the breakpoint and stratum-specific effect estimates**

**Supplementary Table 4. Mixed-effects logistic regression analysis of the association between SIRI and vascular crisis risk after PSM**

**Supplementary Figure 1. Covariate balance before and after PSM**

| **Supplementary Table 1. Baseline characteristics of patients stratified by vascular crisis status following finger replantation** | | | | |
| --- | --- | --- | --- | --- |
| **Characteristic** | **N = 1,744** | **Non-Vascular crisis** | **Vascular crisis** | ***P*-value** |
|  |  | **N = 1,642** | **N = 102** |  |
| **Gender** |  |  |  | 0.502 |
| Male | 1,412 (80.96%) | 1,332 (81.12%) | 80 (78.43%) |  |
| Female | 332 (19.04%) | 310 (18.88%) | 22 (21.57%) |  |
| **Age (y)** | 41 (31, 51) | 41 (31, 51) | 42 (32, 50) | 0.990 |
| **Smoke** |  |  |  | <0.001 |
| No | 1,413 (81.02%) | 1,352 (82.34%) | 61 (59.80%) |  |
| Yes | 331 (18.98%) | 290 (17.66%) | 41 (40.20%) |  |
| **Hypertension** |  |  |  | <0.001 |
| No | 1,615 (92.60%) | 1,536 (93.54%) | 79 (77.45%) |  |
| Yes | 129 (7.40%) | 106 (6.46%) | 23 (22.55%) |  |
| **Diabetes** |  |  |  | 0.694 |
| No | 1,714 (98.28%) | 1,614 (98.29%) | 100 (98.04%) |  |
| Yes | 30 (1.72%) | 28 (1.71%) | 2 (1.96%) |  |
| **Ischemic time (h)** | 5.05 (4.02, 6.39) | 5.07 (4.02, 6.40) | 4.90 (3.93, 6.28) | 0.701 |
| **Duration of surgery (h)** | 3.00 (2.20, 4.50) | 3.00 (2.20, 4.40) | 3.30 (2.50, 4.50) | 0.177 |
| **Preservation method** |  |  |  | <0.001 |
| Dry at low temperature | 413 (23.68%) | 6 (0.37%) | 1 (0.98%) |  |
| Dry at room temperature | 7 (0.40%) | 1,286 (78.32%) | 38 (37.25%) |  |
| Contact with liquid | 1,324 (75.92%) | 350 (21.32%) | 63 (61.76%) |  |
| **Complication** |  |  |  | 0.955 |
| No | 1,467 (84.12%) | 1,381 (84.10%) | 86 (84.31%) |  |
| Yes | 277 (15.88%) | 261 (15.90%) | 16 (15.69%) |  |
| **Seniority** |  |  |  | <0.001 |
| Trainee | 1,282 (73.51%) | 420 (25.58%) | 42 (41.18%) |  |
| Specialist | 462 (26.49%) | 1,222 (74.42%) | 60 (58.82%) |  |
| **Injury finger** |  |  |  | 0.331 |
| Thumb | 293 (16.80%) | 274 (16.69%) | 19 (18.63%) |  |
| Index | 448 (25.69%) | 420 (25.58%) | 28 (27.45%) |  |
| Middle | 283 (16.23%) | 271 (16.50%) | 12 (11.76%) |  |
| Ring | 204 (11.70%) | 197 (12.00%) | 7 (6.86%) |  |
| Little | 159 (9.12%) | 150 (9.14%) | 9 (8.82%) |  |
| Multidigit | 357 (20.47%) | 330 (20.10%) | 27 (26.47%) |  |
| **Injury number** |  |  |  | 0.170 |
| 1 | 1,385 (79.42%) | 1,311 (79.84%) | 74 (72.55%) |  |
| 2 | 223 (12.79%) | 207 (12.61%) | 16 (15.69%) |  |
| ≥3 | 136 (7.80%) | 124 (7.55%) | 12 (11.76%) |  |
| **Injury level** |  |  |  | 0.335 |
| Tamai I | 155 (8.89%) | 150 (9.14%) | 5 (4.90%) |  |
| Tamai II | 262 (15.02%) | 247 (15.04%) | 15 (14.71%) |  |
| Tamai III | 521 (29.87%) | 495 (30.15%) | 26 (25.49%) |  |
| Tamai IV | 311 (17.83%) | 293 (17.84%) | 18 (17.65%) |  |
| Tamai V | 308 (17.66%) | 285 (17.36%) | 23 (22.55%) |  |
| Multiple level | 187 (10.72%) | 172 (10.48%) | 15 (14.71%) |  |
| **Injury mechanism** |  |  |  | 0.015 |
| Chainsaw injury | 159 (9.12%) | 142 (8.65%) | 17 (16.67%) |  |
| Crush injury | 874 (50.11%) | 826 (50.30%) | 48 (47.06%) |  |
| Avulsion injury | 377 (21.62%) | 352 (21.44%) | 25 (24.51%) |  |
| Clean cut injury | 334 (19.15%) | 322 (19.61%) | 12 (11.76%) |  |
| **Vein graft** |  |  |  | 0.032 |
| No | 1,702 (97.59%) | 1,606 (97.81%) | 96 (94.12%) |  |
| Yes | 42 (2.41%) | 36 (2.19%) | 6 (5.88%) |  |
| **Degree of detachment** |  |  |  | 0.786 |
| No | 64 (3.67%) | 60 (3.65%) | 4 (3.92%) |  |
| Yes | 1,680 (96.33%) | 1,582 (96.35%) | 98 (96.08%) |  |
| **Bone and joint** |  |  |  | 0.346 |
| Syntripsis | 467 (26.78%) | 446 (27.16%) | 21 (20.59%) |  |
| Fracture involving the joint | 406 (23.28%) | 380 (23.14%) | 26 (25.49%) |  |
| Transverse fracture | 871 (49.94%) | 816 (49.70%) | 55 (53.92%) |  |
| **Nerve injury** |  |  |  | 0.597 |
| Fingertip nerve injury | 345 (19.78%) | 329 (20.04%) | 16 (15.69%) |  |
| Crush injury of digital nerve | 663 (38.02%) | 626 (38.12%) | 37 (36.27%) |  |
| Cut injury of digital nerve | 419 (24.03%) | 391 (23.81%) | 28 (27.45%) |  |
| Avulsion injury of digital nerve | 317 (18.18%) | 296 (18.03%) | 21 (20.59%) |  |
| **Tendon injury** |  |  |  | 0.078 |
| None | 933 (53.50%) | 890 (54.20%) | 43 (42.16%) |  |
| Zone I | 346 (19.84%) | 322 (19.61%) | 24 (23.53%) |  |
| Zone II | 429 (24.60%) | 397 (24.18%) | 32 (31.37%) |  |
| Zone III | 10 (0.57%) | 8 (0.49%) | 2 (1.96%) |  |
| Zone IV | 8 (0.46%) | 8 (0.49%) | 0 (0.00%) |  |
| Zone V | 18 (1.03%) | 17 (1.04%) | 1 (0.98%) |  |
| **Atery number** |  |  |  | 0.013 |
| 1 | 154 (8.83%) | 150 (9.14%) | 4 (3.92%) |  |
| 2 | 1,234 (70.76%) | 1,167 (71.07%) | 67 (65.69%) |  |
| ≥3 | 356 (20.41%) | 325 (19.79%) | 31 (30.39%) |  |
| **Vein number** |  |  |  | 0.054 |
| None | 10 (0.57%) | 10 (0.61%) | 0 (0.00%) |  |
| 1 | 214 (12.27%) | 208 (12.67%) | 6 (5.88%) |  |
| 2 | 370 (21.22%) | 352 (21.44%) | 18 (17.65%) |  |
| 3 | 219 (12.56%) | 199 (12.12%) | 20 (19.61%) |  |
| 4 | 416 (23.85%) | 395 (24.06%) | 21 (20.59%) |  |
| ≥5 | 515 (29.53%) | 478 (29.11%) | 37 (36.27%) |  |
| **Arteriovenous ratio** |  |  |  | 0.435 |
| 1:00 | 11 (0.63%) | 11 (0.67%) | 0 (0.00%) |  |
| 1:01 | 113 (6.48%) | 110 (6.70%) | 3 (2.94%) |  |
| 1:02 | 170 (9.75%) | 157 (9.56%) | 13 (12.75%) |  |
| 2:01 | 120 (6.88%) | 116 (7.06%) | 4 (3.92%) |  |
| 2:02 | 368 (21.10%) | 345 (21.01%) | 23 (22.55%) |  |
| ≥2：3 | 962 (55.16%) | 903 (54.99%) | 59 (57.84%) |  |
| **Atery injury** |  |  |  | 0.022 |
| Fingertip artery injury | 342 (19.61%) | 323 (19.67%) | 19 (18.63%) |  |
| Crush injury of digital artery | 656 (37.61%) | 623 (37.94%) | 33 (32.35%) |  |
| Cut injury of digital artery | 413 (23.68%) | 389 (23.69%) | 24 (23.53%) |  |
| Avulsion injury of digital artery | 293 (16.80%) | 275 (16.75%) | 18 (17.65%) |  |
| Avulsion injury of digital artery with defect ≥1.5cm | 40 (2.29%) | 32 (1.95%) | 8 (7.84%) |  |
| **Skin injury** |  |  |  |  |
| Nail bed injury | 526 (30.16%) | 500 (30.45%) | 26 (25.49%) |  |
| Nail bed and dorsal finger skin injury | 715 (41.00%) | 681 (41.47%) | 34 (33.33%) |  |
| Crush injury of dorsal finger skin injury | 171 (9.81%) | 157 (9.56%) | 14 (13.73%) |  |
| Cut injury of dorsal finger skin injury | 187 (10.72%) | 173 (10.54%) | 14 (13.73%) |  |
| Avulsion injury of dorsal finger skin injury | 123 (7.05%) | 115 (7.00%) | 8 (7.84%) |  |
| Avulsion injury with defect of dorsal finger skin | 22 (1.26%) | 16 (0.97%) | 6 (5.88%) |  |
| **Flap** |  |  |  | <0.001 |
| No | 1,656 (94.95%) | 1,572 (95.74%) | 84 (82.35%) |  |
| Yes | 88 (5.05%) | 70 (4.26%) | 18 (17.65%) |  |

| **Supplementary Table 2. Optimal cutpoint analysis of SIRI for predicting vascular crisis** | | | | | | | | | | | | | | | |
| --- | --- | --- | --- | --- | --- | --- | --- | --- | --- | --- | --- | --- | --- | --- | --- |
| **Risk factors** | **AUC** | **Direction** | **Cutpoint** | **youden** | **Sensitivity** | **Specificity** | **PPV** | **NPV** | **Accuracy** | **Precision** | **Recall** | **TP** | **FP** | **TN** | **FN** |
| **SIRI** | 0.695 | ≥ | 1.214 | 0.318 | 75.50% | 56.30% | 9.70% | 97.40% | 57.40% | 9.70% | 75.50% | 77 | 718 | 924 | 25 |

| **Supplementary Table 3. Threshold effect analysis of SIRI on vascular crisis using standard and piecewise logistic regression models, with identification of the breakpoint and stratum-specific effect estimates** | | |
| --- | --- | --- |
|  | **adjusted OR (95% CI)** | ***P*-value** |
| **Fitting by standard Logistic regression model** | 1.26 (1.12, 1.42) | <0.001 |
| **Fitting by piecewise Logistic regression model**  **(Break-Point = 3.388)** |  |  |
| SIRI < 3.388 | 2.00 (1.45, 2.77) | <0.001 |
| SIRI ≥ 3.388 | 0.91 (0.67, 1.24) | 0.564 |
| **Log likelihood ratio** |  | 0.001 |
| Adjusted for: sex, age, ischemic time, preservation method, complication, duration of surgery, seniority, smoke, hypertension, diabetes, vein graft, degree of detachment, bone and joint, flap and non-calculated laboratory parameters | | |

| **Supplementary Table 4. Mixed-effects logistic regression analysis of the association between SIRI and vascular crisis risk after PSM** | | | | | |
| --- | --- | --- | --- | --- | --- |
| **Characteristic** | **N** | **Event N** | **OR** | **95% CI** | ***P*-value** |
| **SIRI** | 1,446 | 76 | 2.65 | 1.81, 3.88 | <0.001 |
| **subclass.sd** | 1,446 | 76 | 7.99 |  |  |
| Abbreviations: CI = Confidence Interval, OR = Odds Ratio | | | | | |
